# Reflections and experiences of a co-researcher involved in a renal research study

**DOI:** 10.1186/s40900-018-0120-4

**Published:** 2018-10-29

**Authors:** Sue Marks, Elspeth Mathie, Jane Smiddy, Julia Jones, Maria da Silva-Gane

**Affiliations:** 1 Public Involvement in Research group (PIRg), CRIPACC, University of Hertfordshire, Hatfield, AL10 9AB UK; 2 CRIPACC, University of Hertfordshire, Hatfield, AL10 9AB, UK; 3 Department of Nursing, Midwifery and Health, Faculty of Health and Life Sciences, Northumbria University, Newcastle upon Tyne, NE7 7XA UK; 4 Lister Hospital, Stevenage, East and North Herts NHS Trust, Stevenage, UK

**Keywords:** Patient and public involvement, Renal research, Co-researcher

## Abstract

**Background:**

Patient and Public Involvement (PPI) is seen as a prerequisite for health research. However, current Patient and public involvement literature has noted a paucity of recording of patient and public involvement within research studies. There have been calls for more recordings and reflections, specifically on impact. Renal medicine has also had similar criticisms and any reflections on patient and public involvement has usually been from the viewpoint of the researcher. Roles of patient and public involvement can vary greatly from sitting on an Advisory Group to analysing data. Different PPI roles have been described within studies; one being a co-researcher. However, the role of the co-researcher is largely undefined and appears to vary from study to study.

**Methods:**

The aims of this paper are to share one first time co-researcher’s reflections on the impact of PPI within a mixed methods (non-clinical trial) renal research study. A retrospective, reflective approach was taken using data available to the co-researcher as part of the day-to-day research activity. Electronic correspondence and documents such as meeting notes, minutes, interview thematic analysis and comments on documents were re-examined. The co-researcher led on writing this paper.

**Results:**

This paper offers a broad definition of the role of the co-researcher. The co-researcher reflects on undertaking and leading on the thematic analysis of interview transcripts, something she had not previously done before. The co-researcher identified a number of key themes; the differences in time and responsibility between being a co-researcher and an Advisory Group member; how the role evolved and involvement activities could match the co-researchers strengths (and the need for flexibility); the need for training and support and lastly, the time commitment. It was also noted that it is preferable that a co-researcher needs to be involved from the very beginning of the grant application.

**Conclusions:**

The reflections, voices and views of those undertaking PPI has been largely under-represented in the literature. The role of co-researcher was seen to be rewarding but demanding, requiring a large time commitment. It is hoped that the learning from sharing this experience will encourage others to undertake this role, and encourage researchers to reflect on the needs of those involved.

## Lay summary

There is limited reporting of patient and public involvement (PPI) within research studies and its impact, particularly in renal research. There are few reports from the perspective of lay research team members.

When designing the PIPPIN project (Patients as Partners in Promoting Shared Care), the researchers recognised that PPI had a significant role to play in its design and conduct. To bring an alternative viewpoint to the study, they recruited a first-time lay co-researcher. She was an equal partner within the research team, who carried out some or all of the research activities alongside or independent of the academic researchers.

The co-researcher was recruited from the well established Public Involvement in Research Group (PIRg) at the Centre for Research in Public Health & Community Care (CRIPACC), University of Hertfordshire.

The activities and impact of PPI is often measured or evaluated from the researcher perspective. In this paper, the co-researcher outlines, reflects on, and critically evaluates her activities at the different stages of the research cycle, and its impact on the research outcome. She describes how her role evolved, the practicalities and challenges of her involvement and reflects on that involvement and the impact of this PPI contribution. Her reflections, retrospective and based on her overall experience, were written at the project’s completion.

We hope that the co-researcher’s observations on her role, and the lessons learnt, will be useful for researchers aiming to recruit a PPI co-researcher to their team, and for other members of the public considering undertaking a similar role.

## Background

Involving patients and members of the public in health research is a now a prerequisite by most funders of research in the UK [1]. Patient and Public Involvement (PPI) is viewed as improving research, resulting in studies which are more relevant and useful to the public [2]. There is also a moral and ethical argument that patients should be involved as they are the end users of health research [3].Several authors have described the lack of reporting of PPI within research studies [4] and the lack of evidence base on the impact of PPI [2, 5]. One health area which is seen to have limited reporting of PPI is renal research [6], Morris et al. (2017) commented that “few studies in renal research report on PPI and the impact this may have had on research design” (p.23) [7] although examples do exist [8].

In this paper, a first-time lay co-researcher details the experiences of her involvement in a non-clinical trial renal research project and the impact of this PPI contribution. The research team have encouraged and supported her to write up her reflections which we hope will be useful to other members of the public considering undertaking a co-researcher role. Public involvement in research is defined as “research being carried out ‘with’ or ‘by’ members of the public rather than ‘to’, ‘about’ or ‘for’ them” [9] and in health research this includes a variety of different people (carers, patients, public). Fredriksson and Tritter (2017) [10] make the distinction between patient and public in PPI. Firstly, people with direct experience of health conditions either themselves or through a member of the family and secondly, people who have a more general interest in health and bring a ‘public’ view to health research. In this study, the co-researcher was recruited from the latter category. The distinction between these two types of PPI contributors and what impact they may have has been under explored. Public involvement can be on a number of levels including consultation and citizen control [11]. INVOLVE (a National Advisory Group support active public involvement in National Health Service, public health and social care research) describes different PPI approaches: consultation, collaboration and service-led research [9]. PPI contributors can be involved at different stages of the research cycle, have different roles and carry out different activities within a research project.

PPI contributors are now acting in roles which were formerly only carried out by researchers; co-applicants and co-researchers. A co-researcher is distinct to a “user researcher” who “is someone who uses or has used health and/or social care services because of illness or disability, who is also a researcher” [12] and it is usually assumed they are researching their own health condition. Participatory research also uses the term co-researcher but can refer to participants being involved in the research process [13]. The definition of a co-researcher in the context of PPI fits with the growing enthusiasm for co-design and co-production [14]. The PPI ‘co-researcher‘role within research studies has been described in a number of different health conditions settings; cancer [15], dementia [16], with older people [17] and children [18, 19]. Two recent studies which focused on PPI involved co-researchers in interviewing and data analysis [20, 21].

To our knowledge, there is no accepted definition of the role of a co-researcher and it is suggested that each study has different interpretations of what these co-roles involve [22]. Bindels and colleagues [23] offer one definition of older co-researchers as “older people who collaborate on an equal basis with academic researchers in research teams” (p.3) . This suggests that the co-researcher role requires more involvement than being on an Advisory Group or part of a PPI panel [24]. Our working definition is “co-researchers are equal partners of the research team and carry out some or all of the research activities alongside or independent of the academic researchers”. We acknowledge that researchers and PPI contributors may have different expectations of the co-researcher role and sometimes the role might be the same as a researcher (carrying out the same tasks) or limited to quite distinct tasks. Furthermore, it is unknown what the impact is when there is a co-researcher involved in a study compared to a team solely comprised of academic researchers. To what extent the input from co-researchers compliment or differ from academic researchers has had limited attention. This paper describes the practicalities of being involved in a research project from the viewpoint of the co-researcher (SM) and reflects on the impact of that involvement.

### The research study

The project that is described in this paper is the PIPPIN project; ‘Patients as Partners in Promoting Shared Care -what models work best to improve patient experience in long term conditions services?' Ethics approval was granted in October 2014 by NRES committee London - City and East. The mixed method (non-clinical) study was conducted within two renal medicine units in the East of England and had over 150 participants overall; including a quantitative questionnaire with patients and qualitative interviews with patients, NHS nurse managers, dieticians, nurse practitioners educators, renal managers and doctors. The main findings of this study will be covered in a forthcoming publication.

## Methods

The focus of this paper is a personal reflection of the co-researcher on her involvement in the research. The writing of this article has been guided by the GRIPP2 checklist that has been developed to improve the reporting of PPI within a research study and associated publications [25]. Our paper is structured and submitted as a research paper as we consider the topic and reflective approach to be worthy of a full article, rather than a shorter report or letter to the editor. This new style of paper is intended to generate discussion and it is suggested that additional publication categories in journals may be required as the reporting and reflections on PPI in research increases.

In preparing for writing this paper, the co-researcher looked back through emails and other correspondence with the researchers. In addition, her involvement was documented through copies of research meeting notes, minutes of formal Advisory Groups, data analysis coding themes suggested by the co-researcher, track-changes on documents (including abstracts, interview questions, newsletters, reports to funders) and presentations. Her reflections are retrospective and based on her overall experience at the end of the project. The impact of involvement is usually measured or evaluated from the researcher perspective [26] although PPI reflections have also been included [24, 27, 28]. In this paper, the co-researcher critically evaluates the impact of her own involvement. The systematic review by Brett, Staniszewska, Mockford et al. (2014) [2] discusses reporting on the impact on the co-researcher as well as touching on the impact on the research.

This paper concentrates on the views of the co-researcher and firstly discusses the different PPI roles within the research study, secondly, the co-researcher’s involvement throughout the research cycle and lastly, overall reflections and challenges.

## Results

### Patient and public involvement roles

The Centre for Research in Public Health & Community Care (CRIPACC) at the University of Hertfordshire has a long history of involving public members in research. Recognising that patients, carers, and the public have a significant role to play in the design and conduct of projects, CRIPACC established a group to promote their involvement. The Public Involvement in Research Group (PIRg) was established in 2005 to bring lay voices and opinions to studies. An honorarium is available to the PIRg members to compensate for time spent attending regular meetings and reading/commenting on documents. The Group has a core membership of around 15 who bring a wide range of experiences from their working and personal lives, and carer responsibilities. Researchers present their research ideas, proposals, documents to the PIRg either in person, by email or at a PIRg meeting, and call for volunteers to become involved at various stages of the research cycle [20, 24, 29].

The PIPPIN research team had previously collaborated with the PIRg in numerous research projects, and appreciated the benefits of the members’ contributions. When developing the PIPPIN study bid, the lead researcher believed it essential that the research design reflected meaningful active patient and public involvement. The study was discussed at PIRg meetings, and some of its members acted as a reference group and guided the research protocol and the lay summary during the early stages. The PIPPIN study built upon a previous study – SELFMADE – self-management of renal patients [30] which had involved the co-researcher as an Advisory Group member. The co-researcher first heard about PIPPIN study at a SELFMADE workshop. One example of her contribution to SELFMADE was to suggest that patients with experience of dialysis become mentors for those who were struggling with their dialysis treatment. This resulted in a new paid post being created – a Peer Support Facilitator – and one criteria for the role is that they are a renal patient.

Funding was included in the grant bid for a lay member to be recruited as a co-researcher with the view to becoming an integral part of the small research team. The lay co-researcher, recruited from the PIRg membership, is not an academic staff member but holds a contract with the University as an “Expert by Experience” to participate in research projects, providing a lay person’s perspective. The co-researcher received payment for her time spent on the study and travel expenses, but sees herself primarily as a volunteer.

Patient and Public Involvement within this project had a number of components to enable a breadth of opinion and input: initial PIRg input in study design and then once the study was funded further PPI input through a reference group, a separate Advisory Group and a co-researcher. Members of the PIRg were involved as a reference group, a small virtual group of 7 members (4 PPI members, 2 academics and one medical consultant) which gave input in relation to specific aspects of the research design. Activities focused on advising on the research protocol and data collection tools and reviewing of emerging findings from the scoping exercise and case studies over the period of the research study. The project also had a separate Advisory Group (4 PPI members (including the co-researcher and representatives from local renal Patient Associates/Patient Advocates, National Kidney organisations), 2 medical consultants, one counsellor, 4 academics plus research team) with six monthly face to face meetings as well as on-going electronic communications. There was some overlap of membership between these two groups but they had distinct aims. The Advisory Group’s role was to monitor and advise on the progression of the study, whilst the role of the smaller reference group was used ad hoc to input into specific research activities and the mode of access was a shorter time scale (via email). These involvement activities fed into decision-making relating to the project.

In this study, the co-researcher was a retired member of the public whose whole professional communications/policy career was in the health field. The co-researcher had been a PIRg member since 2010, had contributed to numerous stages of research projects during her time with the PIRg, but never at co-researcher level. The co-researcher had no personal or professional experience of the treatment of patients with renal disease, and therefore brought an alternative viewpoint to the study. She brought complementary skills from a public perspective and her experience included communicating with NHS staff and patients and “basic common sense”.

The new project was discussed at a PIRg meeting and it was decided that she would be the most suitable due to the knowledge she had gained as an active member of the previous SELFMADE Advisory Group (Renal Study). The co-researcher role presented an opportunity for further development and was a continuation of a similar project and setting. The PIRg are offered PPI opportunities and attempts are made to best use people’s skills and make sure everyone gets an opportunity to get involved.

The co-researcher reflects on the differences between the two roles of an Advisory Group member (previous studies) and co-researcher (present study):

*“I had been a member of advisory or steering groups on various studies, attending and participating in meetings, commenting on documents, and offering guidance. I had limited responsibilities. I read and commented on documents and offered advice. Taking on the role of co-researcher meant additional responsibilities and time commitment.”* (Reflective note)

*“I wanted to take on the role of co-researcher so I could immerse myself in a study throughout most of its stages, and not just dip in and out during its progress. I had no personal or professional experience of renal disease, so I brought a different viewpoint to the research*. *As a first-time co-researcher, I was unsure of what was expected of me but I was mentored and trained by the lead researchers and constructive feedback was given at every stage.”* (Reflective note)

There was no role description when the co-researcher joined the project;

*“When recruited, there was no role profile of what is expected of a lay co-researcher, and no guidance on the activities I would be expected to take part in. I was familiar with the research cycle, but needed to be briefed on the anticipated pathway of my role in this project.”* (Reflective note)

*“Mentoring from the research team was necessary at the beginning of each stage so that I could receive training, where appropriate, to deliver expectations. Following training and one-to-one meetings, and as the study progressed, I established a good working relationship with the researchers and the research team. I became more “embedded” in the research objectives, and my knowledge and confidence increased, as did my contributions.”* (Reflective note)

### Co-researcher involvement during the research cycle

Using the INVOLVE research cycle as a guide [9], the co-researcher has reflected on her involvement and engagement over the course of the research project. Table 1 details research activities over the course of the study. She particularly noticed that her level of input within the research team varied at each stage of the study, sometimes being collaborative, sometimes consultative and other times led by the co-researcher. Her reflections continued during the course of writing this article.

**Table 1:** Timeline of co-researcher’s key activities

May 2014	First became involved with this project when the lead researcher asked me to take a look at the protocol which was in its final draft.
By July 2014	Recruited as co-researcher
July 2014	Made useful contributions to the research protocol. Contributed to the lay summary.
September 2014	Commented on interview schedules
November 2014	Contributed to discussions at the Advisory Group Meeting on how to capture costs for the project.
February 2015	Commented on the lay summary which was submitted to the funders. The abstract for the British Kidney Association (BKA) conference.
July 2015:	Had meeting with lead researcher who gave a demonstration of the SPSS stats system. Was not involved with inputting the stats.
December 2015	Data analysis of 33 interview transcripts started and continued until January 2017
March/April 2016	Submitted comments/suggestions for questions for 2nd round of interviews. Summary of interview themes sent to research team.
March 2016	Commented on Patient Research Newsletter text.
October 2016	Commented on abstract for Health Symposium.
October 2016	Commented on 3rd round questions, & suggested new/alternative questions.
December 2016	Final end date of study. Dissemination continues into 2017 and beyond. Commented on BRS conference abstract
January 2017	Commented on the report to the funder
March 2017	Commented on poster presentation for the British Renal Society conference
*Activities Throughout The Research*	
Communication: Emails, replying/dealing with emails, queries, documents.	
Meetings (face to face): Participated in 7 Research Team Meetings, Advisory Meetings, plus numerous one-to-one meetings and email exchanges regarding training and the progress/development of the study.	
Document Review: commented on/edited over 10 draft documents, (in addition to analysing the interview transcripts); these included: the research protocol, the patient questionnaire findings, patient information newsletters, interview schedules, lay summary, abstract, submissions to funder, scoping report, funder project report, presentations.	
Dissemination: lead author of this article, commented on abstract for Health Research Symposium 2017, abstract and poster for British Renal Society (BRS) conference.	

Please note that this list is not comprehensive as the co-researcher did not keep a running log of involvement. The table above was compiled by revisiting documents and email exchanges

*“My role evolved over the period of the study. I was given the opportunity to opt in or out of involvement with each activity, but took on more of the workload and responsibilities as time went on.”* (Reflective note)

During the period of the study, the co-researcher attended formal meetings at the University, plus regular one-to-one meetings/email exchanges with the lead researchers to discuss progress and for training purposes. Except for formal meetings, the majority of her activities were undertaken at home rather than travelling to the University.

*“The team were considerate of my other work and social commitments and organised meetings to coincide with my availability. My parking arrangements were arranged and they fed me if the meeting was over the lunch period. However, the uncertainty of when I was going to be called upon to take part in an activity was difficult for me. For example, on some occasions, deadlines were tight and I was asked to complete a task shortly before a holiday or when I was working on other projects.”* (Reflective note)

#### Idea/concept of the research

The concept of the project was already in place by the time the co-researcher was recruited, so there was little opportunity for her to assist in developing the research topic. It was also the case that the research bid and funding were in place before the current Principal Investigator (MSG) and lead researcher (JS) were appointed, so none of the research team were involved at this stage.

However, as the plan and design evolved in the early stages, and the co-researcher became more familiar with the issue and research objectives, she contributed increasingly to the discussions on the design with both the researchers at one-to-one meetings, and meetings of the Advisory Group and research team. As her experience of research methodology was not extensive, her contribution was limited in the early stages. The researchers spent time, in informal training sessions, explaining the different research methods where the training could be tailored to her needs and interest.

#### The grant application

This had already been more or less finalised before the co-researcher was recruited, and she was not a co-applicant for the study. She had an opportunity to comment on the final draft but, not having been involved with earlier discussions, the contribution was minimal. At this time there was also a change in Principal Investigator and recruitment of the lead researcher.

#### Advisory group

The co-researcher was an active member of the research team and attended the Advisory Group meetings in this capacity. This provided an opportunity to contribute to discussions but also, importantly, added to her knowledge base through hearing first-hand the inputs from health professionals and service users. The Advisory Group had terms of reference which included providing advice and guidance and keeping the project to time. The co-researcher saw her role on the Advisory Group as one of providing an additional PPI perspective and being part of the research team enabled her to provide informed contributions from her interpretation of the data.

#### Writing and editing documents

The co-researcher commented on, edited and developed numerous documents throughout the research cycle, including the lay summaries, the interview schedule*,* patient information newsletters, the detailed protocol, reports of progress and drafts of papers:

“*Communications, both written and oral, is one of my key skills and I commented on/edited most of the written documentation to ensure clarity and patient-focus. For example, my contributions to identifying themes to pursue, and framing the interview questions resulted in more meaningful responses from patients and staff.”* (Reflective note)

#### Patient, staff and manager interviews

All the interviews took place within busy hospital renal units where space was limited and were undertaken by academic researchers who had Good Clinical Practice (GCP) training and NHS research passports. For these reasons, the co-researcher was not involved in recruiting participants or the interviews but she remained closely involved at each stage of this process. She was involved in developing the interview guides, identified the topics to pursue, added clarity to the questions avoiding jargon, and suggested alternative and additional questions.

*“I became more familiar with, and knowledgeable about the research topic and the methodology being used as the research stages evolved. I was then able to make more meaningful contributions to discussions and written documentation. It was a very fast learning curve.”* (Reflective note)

#### Data analysis

A Patients Assessment Chronic Illness Care (PACIC) survey [31] was used (which has set questions and is a validated generic tool which is widely available) to ascertain patients’ experiences in each of the two centres. As part of her training, the co-researcher had a brief introduction to the SPSS system (a Statistical Software Package) but it was neither feasible or a constructive use of time for her to contribute to data input from the questionnaires.

*“It would have been too time-consuming for me to work on the data input as it would have meant travelling to the University in addition to time spent on the computer.”* (Reflective note)

However, the introduction to SPSS enabled the co-researcher further understanding of data entry and the research process.

An initial task was to identify and discuss the main findings arising from the quantitative element of the research. Although not involved with analysing the quantitative aspects of the study, the co-researcher led on the analysis of topics raised from the patient survey which contributed to identifying the coding headings for the qualitative analysis of the interviews.

Patients on dialysis at the renal units, staff and managers were interviewed at least twice during the case study period of 12–18 months. The interviews and focus groups were recorded, transcribed and anonymous transcripts distributed to the researchers and co-researcher.

It was at this stage that the co-researcher took the lead in the qualitative data analysis, the first time she had undertaken this activity. The researchers and co-researcher had discussions about qualitative analysis and the co-researcher was able to highlight themes she felt were important from a user perspective. This resulted in stronger patient focussed perspectives being identified.

*“I offered to be the key person to analyse the transcripts from the interviews with patients, staff and management at the two centres. Reading each of the transcripts of the patient and staff first interviews, and focus groups, I identified the issues raised, categorising them into key themes and identifying the positives and challenges of the various aspects of shared care experiences.”* (Reflective note)

The co-researcher produced summaries at the end of the three interview rounds analysing the themes, including references to interesting quotes which substantiated the analyses. Interim summaries were emailed regularly to the research team prior to completing the review of all the transcripts, with an updated final version distributed once the transcripts from that stage had all been read and analysed.

The co-researcher’s thematic analyses were discussed at research team meetings, with others contributing their own comments having read the transcripts. The analyses also enhanced the questions for the second and third round of interviews. It is considered that the analyses and summaries yielded a more focused, patient centred perspective for the interviews, and contributed to more informed group discussions.

Themes were also identified from the initial interviews with nurse managers and focus groups with nursing staff. It became clear that the staff perceptions were of multiple definitions of patient involvement – shared care, self care, self management, partnership management. The co-researcher contributed to team discussions on these findings.

*“By the time this stage was reached, I felt more confident with my knowledge of the research objectives, and led the thematic analysis of the interview/focus group transcripts for each of the interviews with patients, staff and management. This activity played to my strengths, though posed a challenge for me initially as I had no previous experience of this task. It was also time-consuming.”* (Reflective note)

The themes identified by the co-researcher were then transferred by the researchers to NVIVO, the qualitative software computer package used to organise the interview data.

*“My work on analysing the transcripts and identifying relevant issues and quotes created more time for the academic researchers to focus on other aspects of the project.”* (Reflective note)

#### Dissemination

There were a number of conferences that occurred during the course of the project and it was suggested that the co-researcher would like to attend to help present findings. The co-researcher was not available at these times but commented on the development of abstracts, presentations and suggested areas for discussion.

### Overall reflections

#### Experience of taking on the co-researcher role

The co-researcher has reflected on this role within this project. Reflections while writing this article gave her a useful insight into the level of her involvement and what she could offer as a research team member of future studies.

*“When recruited, I had been concerned that my involvement might be a token gesture to demonstrate that the study had PPI involvement. I wanted to be an equal partner in the research team, and it quickly became apparent that I was treated as such. However, at times I felt under pressure as the lone PPI member in the study. With hindsight, I should have initiated informal conversations with fellow PIRg members with experiences of being a co-researcher to learn from their experiences of their roles.”* (Reflective note)

She also reported;

*“I was on a steep learning curve as I had no previous experience at co-researcher level ... This role was challenging which, as a retired professional, was appreciated…It was important to me that, throughout the project, I received excellent mentoring and regular feedback regarding my input.”* (Reflective note)

*“The knowledge I’ve gained with the investment in researchers’ time in mentoring/training will result in me having more effective involvement in future research projects. It is very satisfying for me personally to be part of promoting the benefits of renal patients’ involvement in their own care.”* (Reflective note)

#### Co-researcher’s further reflections on the challenges of the role

Table 1 outlined the time commitment of the co-researcher in the study. One of the main reflections from the co-researcher was not being recruited at the beginning of the research idea being developed. INVOLVE [9] recommend that PPI contributors be recruited and involved at the very early stages [32].

Due to the co-researcher’s lack of experience in this role, there was an increased time commitment for both her and the researchers to ensure an overall understanding of the research cycle and terminology. However, this investment in time ensured that contributions at every stage were beneficial to the study’s progress and outcomes.

*“It was my responsibility to ensure that the balance between the research workload and my outside commitments were known to the research team. I therefore alerted them in advance to periods when I knew I would not be available to work on the study – my holiday dates, and other volunteering commitments/diary obligations.”* (Reflective note)

There were changes in the personnel within the research team during the course of the study. This, as well as an extension of the study period from two-years to three was challenging for both the co-researcher and the newly recruited research team members. The extension also limited the co-researcher’s ability to contribute to PPI input on other research projects.

The lay member’s time was limited due to other work and social commitments. Having no previous experience of involvement at this level, she had not taken account of the sometimes inconvenient timing of the workload that would be needed at each stage. Deadlines for comments were often tight which sometimes meant that other work commitments had to be postponed or cancelled.

*“Some uncertainty about workload timing were beyond the control of the research team. One example was that there were delays in transcribing the interview transcripts. This sometimes resulted in me receiving many at once. As each transcript took between 45 minutes to 1.5 hours to study and comment on, the time commitment at times was quite onerous.”* (Reflective note)

Attempts were made to arrange Research Team and Advisory Group meetings at the University around the co-researcher’s availability to enable her to attend. This meant that she was able to contribute to meetings at most stages of the project. Often there were many months between any activities required from the co-researcher.

“*When I was asked to comment on/contribute to documents, or attend meetings, it would take me time to refresh my memory of the issues involved.”* (Reflective note)

“*One of the challenges I had at the study’s completion was that I did not appreciate that I needed to keep a running log of my activities, involvement and feedback. At the completion of the study I had to spend many hours revisiting documents and emails to identify contributions at each stage.”* (Reflective note)

## Discussion

The role of the co-researcher has been described by a number of research studies and a recent systematic review [33] but few publications written by the co-researchers themselves. This paper offers some suggestions in order to define the role of co-researcher, including equal partnerships. The role of the co-researcher is distinct from that of an Advisory Group member in the depth, breadth and nature of the input. The role requires an on-going time commitment throughout the study and often unpredictable intense periods of word load. The range of benefits and challenges of involving co-researchers has been previously outlined in the literature [23, 33, 34] and many of these recurring themes are echoed in this paper. However, this study drew particular attention to ‘uncertainty’ and ‘unpredictable’ time commitments, the challenge of staying up to date with the research and the need to fully record and document PPI activities. It was felt that early involvement was important as it allows for PPI contributors to “feel part of the research and also have a sense of ownership” [9] (p.13) and maximises opportunities to contribute [32].

There are a few important overall reflections on this co-researcher role in this particular study. Firstly, the need for flexibility and supporting the strengths and skills of co-researchers was a key learning from this study. In the co-researcher’s previous employment (before retirement) she had worked in communications and felt very comfortable commenting on and analysing documents and written summaries. She had no experience of data analysis but though her previous experience of research involving renal patients and her interest in reading transcripts she became very skilled at listening to the patient voice and noticing the differences between patients’ and staff views. The benefits to this study was the qualitative data analysis which was led by the co-researcher and really enabled the patient voice to be heard and identified. Secondly, the need to organise meetings and dates around the co-researcher to enable involvement was essential and the research team made sure that as far as possible the co-researcher could attend all the research meetings.

The co-researcher received informal training, as and, when needed on this study. There have been some varied views in the literature whether lay members of the public should or should not be trained [35–37].  Two years after the PIRg was formed in 2005 at the University of Hertfordshire, a three month training programme on research methods was offered. For those PIRg members, such as the co-researcher, who have joined more recently, on-going training sessions are offered on topics which are identified by the group. This research project offered additional individual training, which focussed on particular topics as, and when they were needed (rather than in isolation).

There are a number of limitations to acknowledge; obviously, this is not an objective evaluation and is primarily the view of one person, however, hearing the voice of co-researchers directly is very limited within the academic literature. It must also be acknowledged that the training and support was provided by the research team (not an external trainer) which may have led to some bias.

## Conclusion

The involvement of a co-researcher within the PIPPIN study enabled the academic researchers to have the benefit of a ‘lay’ voice in a number of activities throughout the research. Firstly, documents were more user-friendly and these included interview topic guides and presentation of findings. Secondly, the biggest role for the co-researcher in this project was involvement in the qualitative data analysis which was led by the co-researcher, identifying themes and reminding the academic researchers about the voice of the patient. The co-researcher did not have training in qualitative data analysis but had support to interpret and identify themes within the transcripts in the manner that suited her. Face to face meetings to discuss themes was vital to this process. Lastly, the large amount of time that the co-researcher role requires must not be underestimated.

A reflection from the research team is that as more members of the public take on researcher roles there is a need to highlight the accompanying levels of responsibility for roles. Co-researchers may, for various reasons, become unable to complete their role and therefore the need for flexibility is very important. PPI is still largely a voluntary role without formal contracts and obligations. Whilst the move to encourage more members of the public to be involved as co-researchers is very much welcomed and valuable, there is also a warning that these roles must be properly resourced, supported, with expectations discussed at the outset and ethical issues [38] considered.

The strength of the co-researcher role in this study was in its ability for the role to evolve, and having no set role description allowed for flexibility, however, a clear role description and discussions around expectations of involvement are recommended [8]. Opportunities for input evolved as the project developed and collaborative working relationships became more embedded. One approach to recruiting a co-researcher is that researchers outline very specific research tasks which they want the co-researcher to undertake and through advertising and recruitment a co-researcher with the necessary skills can be found. A second approach (which was used in this study) was to recruit a co-researcher and work with the skills they have already have and help to support them in the roles they feel are interesting and develop their skills further. The need for flexibility has been highlighted in other PPI studies [20], and funding applications need to be able to accommodate this flexibility.
